# Antimicrobial Resistance in Pediatric Infections: Current Status, Challenges, and Future Directions

**DOI:** 10.3390/antibiotics15060617

**Published:** 2026-06-17

**Authors:** Clare Dinh, Keykavous Parang

**Affiliations:** Center for Targeted Drug Delivery, Department of Biomedical and Pharmaceutical Sciences, Chapman University School of Pharmacy, Harry and Diane Rinker Health Science Campus, Irvine, CA 92618, USA; cldinh@chapman.edu

**Keywords:** antimicrobial stewardship, antibiotic resistance, carbapenem resistance, ESBL, multidrug-resistant organisms, MRSA, pediatric infections, pneumococcal conjugate vaccine

## Abstract

**Background/Objectives:** Antimicrobial resistance in pediatric infections presents a worsening global public health challenge, with antimicrobial resistance (AMR) accounting for more than one million deaths annually and disproportionately affecting children younger than 5 years of age. Neonates and critically ill children face heightened risk owing to immature immunity, frequent healthcare exposures, and limited therapeutic options. This review synthesizes evidence on the epidemiology, mechanisms of resistance, clinical outcomes, and management of AMR across the full pediatric age range. **Methods:** PubMed/MEDLINE and Google Scholar were searched for literature from 2014 to 2026 using terms covering antibiotic resistance, pediatric populations, and key pathogens. Approximately 1840 records were screened; 69 sources met all inclusion criteria. A narrative synthesis approach was used, given heterogeneity across study designs and outcomes. **Results:** Extended-spectrum β-lactamase (ESBL)-producing Enterobacterales, carbapenem-resistant pathogens, and methicillin-resistant *Staphylococcus aureus* drive substantial morbidity and mortality in children. Approximately one in five pediatric Gram-negative bloodstream isolates are resistant to third-generation cephalosporins, a phenotype independently associated with a roughly three-fold increase in adjusted mortality. Carbapenem-resistant *Klebsiella pneumoniae* bacteremia carries a 30-day mortality approaching 40%, and isolates in low- and middle-income countries (LMICs) frequently harbor multiple resistance genes. Pneumococcal conjugate vaccine implementation was associated with absolute reductions of 7–11% in the proportion of pediatric pneumococcal isolates that were penicillin-non-susceptible or penicillin-resistant, largely by preventing infections caused by resistant serotypes and by reducing antibiotic selection pressure, rather than through a direct effect on resistance mechanisms; global AMR mortality in children younger than 5 years of age fell by more than 50% between 1990 and 2021. **Conclusions:** Pediatric AMR reflects intersecting microbiological, clinical, and health-system challenges. Priority actions include scaling antimicrobial stewardship programs, expanding access to rapid molecular diagnostics, integrating whole-genome sequencing into surveillance, conducting pediatric-inclusive randomized trials, and deploying vaccines as primary prevention tools, with particular emphasis on LMICs where the burden is greatest.

## 1. Introduction

### 1.1. The Global Burden of Antibiotic Resistance

The Global Burden of Disease (GBD) study group’s first comprehensive AMR estimate, encompassing 23 pathogens, 88 pathogen–drug combinations, 204 countries, and 471 million individual records or isolates, calculated that bacterial AMR was associated with an estimated 4.95 million deaths in 2019, of which 1.27 million were directly attributable to AMR [1]. The follow-up GBD analysis covering 1990–2021 estimated 4.71 million deaths associated with AMR and 1.14 million directly attributable to it in 2021, and forecast that, under business-as-usual assumptions, attributable AMR deaths could reach 1.91 million annually by 2050 [2]. Encouragingly, that same analysis observed that AMR mortality decreased by more than 50% in children younger than five years between 1990 and 2021, an improvement driven largely by infant immunization, infection prevention, and improved access to care, evidence that pediatric AMR is responsive to public health investment [2].

Despite this progress, children remain a uniquely vulnerable population. The World Health Organization has identified AMR as a top global health threat, and pediatric-specific data and interventions remain less developed than in adult medicine [3]. National Australian surveillance demonstrated that children are not simply “little adults” in the AMR era: resistance rates, clinical phenotypes, and mortality patterns differ meaningfully across age groups, and extended-spectrum β-lactamase (ESBL)-producing organisms are associated with disproportionately higher mortality in pediatric than in adult bacteremia [4].

### 1.2. Pediatric Antibiotic Use as a Driver of Resistance

Pediatric antibiotic use, both in volume and in agent selection, is a principal driver of resistance. The first Antibiotic Resistance and Prescribing in European Children (ARPEC) point-prevalence survey covering 73 hospitals worldwide found that, on the survey day, 35.4% of pediatric and 21.8% of neonatal European hospital inpatients were receiving an antibiotic, rising to 43.8% and 39.4%, respectively, in non-European hospitals. Antibiotic use was highest in pediatric hematology-oncology wards (61.3%) and pediatric intensive care units (55.8%) [5]. A subsequent multinational point-prevalence analysis of 23,572 pediatric and neonatal inpatients across 56 countries demonstrated very wide variation in the proportion of WHO Access-class antibiotics prescribed, from 7.8% in China to 61.2% in Slovenia in children, and similarly large variation in Watch-class prescribing [6]. Such variation, far in excess of what differences in case-mix can explain, suggests that prescribing practices remain an important target for antimicrobial stewardship.

In the outpatient setting, a national U.S. analysis estimated that of every 1000 outpatient antibiotic prescriptions, only about 70% were appropriate; sinusitis, suppurative otitis media, and pharyngitis, conditions with overlapping bacterial and viral aetiologies in children, accounted for the largest contributions to inappropriate use [7]. Even short courses of common pediatric antibiotics measurably alter the resistome: in a study of 40 children, 100% harbored amoxicillin-resistant oral bacteria, with the median proportion rising from 2.4% in untreated children to 10.9% in those who had received amoxicillin in the prior three months [8].

### 1.3. Healthcare-Associated and Geographic Risk Factors

Healthcare-associated factors, including invasive devices, prolonged hospitalization, and intensive care exposure, create niches for resistant Gram-negative bacilli. In the Australian pediatric Gram-negative bloodstream infection (GNBSI) cohort, 60% of episodes occurred in children with a central venous catheter, and 71% occurred in children with comorbidities [9]. In a single-center pediatric cohort of 102 children with Gram-negative bloodstream infection in Turkey, urinary catheterization was an independent predictor of mortality (odds ratio 5.68; 95% CI 1.14–28.25) and central venous catheterization an independent predictor of sepsis (odds ratio 2.46; 95% CI 1.10–5.53) [10]. In a single-center Saudi Arabian cohort of pediatric cultures, multidrug-resistant (MDR) organisms were detected in 42% of patients and were associated with eight-fold higher mortality (32.4% vs. 3.9%) compared with non-MDR infections [11].

Marked geographic and socioeconomic disparities exist. The Asia-Pacific region, home to roughly 600 million children, faces a disproportionate of AMR burden with limited microbiological infrastructure and inequitable vaccine coverage [12]. A systematic review of pediatric Gram-negative sepsis in low- and middle-income countries (LMICs) demonstrated very high regional resistance rates. For example, neonatal *K. pneumoniae* resistance to ampicillin reached a median of 94% in Asia and 100% in Africa, and to cephalosporins 84% in Asia and 50% in Africa [13]. The BARNARDS study, a prospective network covering seven LMICs in Africa and South Asia, recovered isolates from 2483 culture-confirmed neonatal sepsis cases out of 36,285 enrolled neonates and showed that *K. pneumoniae* was the leading pathogen, with isolates harboring multiple cephalosporin and carbapenem resistance determinants [14]. In Sub-Saharan Africa, 50% of *Salmonella enterica* serotype Typhi isolates from febrile children in Nairobi were multidrug-resistant, with quinolone-resistance-determining region mutations in 67.6% [15]; and in Damascus, Syria, retrospective pediatric data showed widespread resistance to third-generation cephalosporins and a 16% case-fatality rate [16].

Several recent narrative and systematic reviews have addressed pediatric antimicrobial resistance from focused angles [3], examining emerging pathogens and AMR in pediatric and neonatal sepsis. Mahony and colleagues [17] reviewed multidrug-resistant uropathogens in children, reporting a rising prevalence of ESBL-producing Enterobacterales in pediatric urinary tract infections and a recurrent association with prior antibiotic exposure. Huong and colleagues [12] provided a regional perspective limited to the Asia-Pacific, identifying fragmented microbiological surveillance and inequitable vaccine coverage as principal drivers of the region’s disproportionate burden. Le Doare and colleagues [13] synthesized the literature on Gram-negative resistance in pediatric sepsis in resource-limited settings, documenting very high neonatal resistance to ampicillin and third-generation cephalosporins across Asia and Africa. Stocker and colleagues [18] issued a national guideline focused on neonatal early-onset sepsis, and Winteler and colleagues [19] systematically reviewed perinatal antibiotic stewardship from 2000 to 2022. The WHO global research agenda for AMR in human health, published by Bertagnolio and colleagues [20], set 40 priorities to be addressed by 2030, but did not provide an integrated synthesis of pediatric epidemiology, mechanisms, outcomes, vaccination, and stewardship. The most recent dedicated pediatric overview, by Romandini and colleagues [21], emphasized global emerging threats and near-term forecasting; the present review, in contrast, links contemporary (2024–2026) surveillance directly to intervention evidence, vaccination, and stewardship. The present review differs from the previous ones. First, it integrates surveillance and outcome data published as recently as 2024–2026 across the full pediatric age range rather than within a single sub-population. Its principal contribution, and the basis for its novelty, is this integration: the review explicitly couples epidemiology with intervention evidence, antimicrobial stewardship, vaccination, and rapid molecular diagnostics, and frames the trajectory of pediatric AMR as a system responsive to public health investment.

For this narrative review, PubMed/MEDLINE was searched as the primary indexed database, supplemented by Google Scholar to capture surveillance reports and gray-literature sources not indexed in MEDLINE, covering publications from January 2014 to February 2026; foundational studies predating this window were retained when they provided primary data not superseded by newer work. Eligible records reported original data or systematic syntheses on antimicrobial resistance, mechanisms, clinical outcomes, or interventions in pediatric populations (ages 0–18 years, including neonates), published in peer-reviewed journals or formally indexed surveillance reports, and available in English. From approximately 1840 candidate records, 187 underwent full-text review and 69 met the inclusion criteria; full methodological details, including exclusion criteria, are provided in Section 6.

## 2. Results

### 2.1. Epidemiology of Resistance in Key Pediatric Pathogens

In keeping with the order set out in the Abstract, the synthesized evidence is presented as epidemiology of resistance (Section 2.1), mechanisms of resistance (Section 2.2), clinical outcomes (Section 2.3), and management: comprising vaccination (Section 2.4), antimicrobial stewardship (Section 2.5), and novel therapeutics (Section 2.6). Pathogen-specific findings from the studies cited above are summarized in Table 1. We have summarized the findings for Gram-negative multidrug-resistant organisms, such as ESBL-producing Enterobacterales and Carbapenem-resistant Enterobacterales (CRE), and Gram-positive resistant pathogens, such as methicillin-resistant *Staphylococcus aureus* (MRSA).

#### 2.1.1. Gram-Negative Multidrug-Resistant Organisms

ESBL-producing Enterobacterales, principally *Escherichia coli* (*E. coli*) and *K. pneumoniae*, are the dominant Gram-negative resistance phenotype in children. National U.S. surveillance using the Surveillance Network database analyzed 368,398 pediatric Enterobacterales isolates and showed that the prevalence of third-generation-cephalosporin-resistant (3GCR) phenotypes rose from 1.39% in 1999–2001 to 3.0% in 2010–2011, while ESBL phenotypes rose from 0.28% to 0.92% over the same period, significant increases across all demographic groups, including outpatients [22]. The Australian pediatric GNBSI prospective study (931 episodes in 818 children, 2019–2021) showed that 22% of Enterobacterales isolates were resistant to third-generation cephalosporins, with *bla*_CTX-M-15_ identified in 36% of sequenced ESBL-producing isolates [9]. In a 5-year retrospective cohort in Taiwan, ESBL-producing pediatric uropathogens accounted for 14.1% of culture-positive UTIs, with recent antibiotic exposure and a preterm gestational history identified as significant risk factors for acquisition of ESBL-producing organisms [23].

Similar findings have recently been reported in Latin America. In a multicenter retrospective cohort study involving 1250 pediatric patients hospitalized with bloodstream or urinary tract infections across three referral hospitals in Peru, the prevalence of ESBL-producing Enterobacteriaceae reached 32.9% [30]. Prior antibiotic exposure (aOR 5.82) and the presence of a central venous catheter (aOR 2.10) were identified as major independent risk factors for ESBL infection. Importantly, hospitals with more mature antimicrobial stewardship programs demonstrated a lower likelihood of ESBL-producing infections (aOR 0.85), highlighting the direct relationship between stewardship infrastructure and resistance outcomes in pediatric populations.

Carbapenem-resistant Enterobacterales (CRE) represent an emerging and severe threat. In a Chinese single-center cohort of 70 children with carbapenem-resistant *K. pneumoniae* bacteremia, 30-day mortality reached 39.4% in neonates and 43.2% in older children [25]. In a multinational pediatric oncology and hematopoietic stem-cell transplant cohort, 9% of Gram-negative bloodstream isolates were meropenem-resistant, and prior carbapenem exposure was significantly associated with subsequent resistant infection [26]. The BARNARDS neonatal sepsis network across seven LMICs documented that Enterobacterales isolates, including *K. pneumoniae*, *E. coli*, and *Enterobacter cloacae* complex, harbored multiple cephalosporin and carbapenem resistance genes and that all isolated pathogens were resistant to multiple antibiotic classes, including those used to treat neonatal sepsis [14].

**Figure 1:**
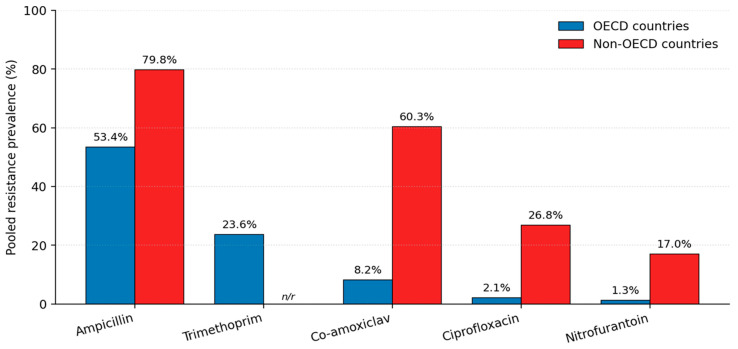
Pooled prevalence of antibiotic resistance in community-acquired pediatric urinary tract infections caused by *E. coli*, comparing OECD and non-OECD countries. This figure was generated by the authors from data reported in [24] and is not reproduced from the original publication. Trimethoprim was not separately reported as a pooled estimate for non-OECD countries in the source meta-analysis. n/r = not reported.

Outside the inpatient setting, a systematic review and meta-analysis of community-acquired pediatric UTIs caused by *E. coli* pooled data from 58 observational studies and 77,783 isolates and demonstrated marked Organization for Economic Co-operation and Development (OECD) versus non-OECD disparities [24]. These findings are summarized graphically in Figure 1.

#### 2.1.2. Gram-Positive Resistant Pathogens

MRSA remains a major pediatric concern. U.S. population-based surveillance documented 876 invasive MRSA infections in pediatric patients during 2005–2010, with 39% occurring in infants and 42% community-associated. The estimated invasive MRSA incidence in 2010 was 43.9 per 100,000 in infants younger than 90 days, roughly 22-fold higher than in older infants and children (2.0 per 100,000), and was disproportionately higher in Black children (6.7 per 100,000) than in other racial groups (1.6 per 100,000); community-associated MRSA incidence rose 10.2% per year over the surveillance period [27]. In multinational pediatric oncology and hematopoietic stem-cell transplant cohorts, methicillin resistance accounted for 17% of *S. aureus* isolates and vancomycin resistance for 40% of *Enterococcus faecium* isolates [26].

In the Australian Pediatric AMR Surveillance Program [4], pediatric *S. aureus*, enterococcal, and Gram-negative bloodstream infections were tracked across 34 hospitals during 2013–2016. In a Turkish single-center pediatric enterococcal-bacteremia cohort, *E. faecium* demonstrated significant ampicillin resistance and an emerging vancomycin-resistant enterococcus (VRE) profile [31].

### 2.2. Mechanisms of Resistance Relevant to Pediatric Practice

β-lactamase production remains the dominant resistance mechanism among Gram-negative pathogens in children. CTX-M-type ESBLs are globally dominant; in the Australian pediatric Gram-negative bloodstream cohort, *bla*_CTX-M-15_ was identified in 36% of sequenced ESBL isolates and *bla*_SHV-12_ in 11% [9]. Carbapenemases (KPC, NDM, OXA-48-like) extend the resistance spectrum to virtually all β-lactam antibiotics and pose particularly severe therapeutic challenges in pediatric care due to the limited number of approved alternative agents [32,33]. Although these resistance mechanisms are not unique to pediatric isolates, their clinical consequences are amplified in children by developmental differences in pharmacokinetics and immunity, a narrower range of approved antimicrobial agents, and frequent exposure to invasive devices, all of which constrain therapeutic options in ways that differ from adult practice.

**Figure 2:**
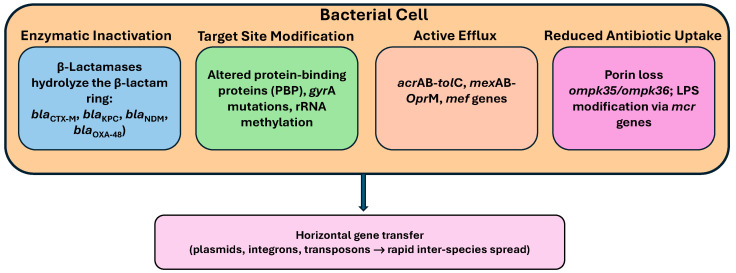
Conceptual schematic of the major mechanisms of antimicrobial resistance encountered in pediatric pathogens [32,33,40,41,42,43,44,45]. The four principal classes include: (i) enzymatic inactivation, dominated by β-lactamases encoded by *bla*_CTX-M_, *bla*_KPC_, *bla*_NDM_, and *bla*_OXA-48_ genes; (ii) target site modification, including altered penicillin-binding proteins encoded by *mecA*, fluoroquinolone resistance through *gyrA* mutations, and macrolide resistance via *erm*-mediated ribosomal RNA methylation; (iii) active efflux mediated by *acrAB-tolC*, *mexAB-oprM*, and *mef* gene systems; and (iv) reduced antibiotic uptake through porin loss (*ompK35*/*ompK36*) or LPS modification via *mcr* genes. All gene names are italicized per standard nomenclature. LPS = lipopolysaccharide; PBP = penicillin-binding protein.

Among Gram-positive pathogens, methicillin resistance in *S. aureus* (mediated by *mecA*-encoded penicillin-binding protein PBP2a), penicillin-binding-protein mutations in *Streptococcus pneumoniae*, *erm*-mediated ribosomal methylation conferring macrolide resistance, and *vanA*/*vanB*-mediated vancomycin resistance in enterococci remain leading clinical concerns [34,35,36,37]. Horizontal gene transfer, mediated principally by conjugative plasmids and integrons [38,39,40], accelerates the inter-species spread of these resistance determinants and underlies many of the regional clonal expansions. The major mechanisms are summarized schematically in Figure 2.

Figure 2 illustrates the key mechanisms of antibiotic resistance observed in pediatric pathogens. The four principal classes of mechanism are: (i) enzymatic inactivation of antibiotics, dominated by β-lactamases (ESBL, KPC, NDM, and OXA-48-like enzymes); (ii) modification of the antibiotic target site (altered penicillin-binding proteins, *gyrA* mutations, and ribosomal RNA methylation); (iii) active efflux of antibiotic from the cell (AcrAB-TolC, MexAB-OprM, and *mef*-encoded systems); and (iv) reduced antibiotic uptake through porin loss (e.g., OmpK35/36) or lipopolysaccharide modification (e.g., *mcr* genes). Resistance determinants encoding these mechanisms are widely disseminated by horizontal gene transfer via plasmids, integrons, and transposons, which underlie the rapid inter-species spread of resistance documented in pediatric Gram-negative surveillance [32,33,40,41,42,43,44,45].

**Figure 3:**
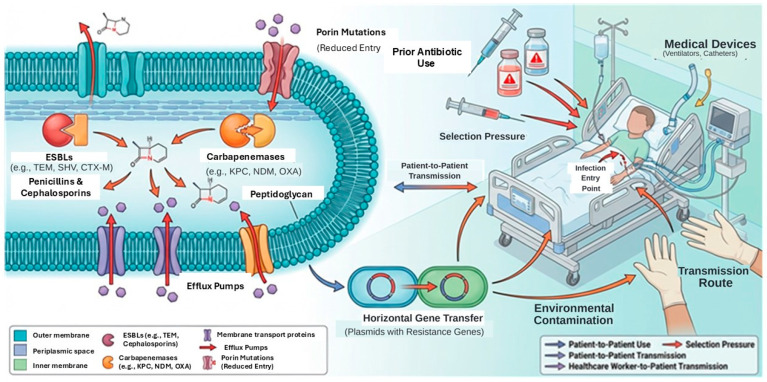
Antibiotic resistance in pediatric infections: mechanisms and hospital risk factors. The left panel illustrates molecular mechanisms of resistance within the bacterial cell membrane, including enzymatic inactivation by *bla*CTX-M, *bla*SHV, *bla*TEM (ESBLs), and *bla*KPC, *bla*NDM, *bla*OXA (carbapenemases), active efflux pump systems, and porin mutations conferring reduced antibiotic uptake; horizontal gene transfer via plasmids disseminates these determinants across species. The right panel depicts hospital-level risk factors and transmission routes that amplify resistance in pediatric settings. **Arrow key: blue arrows**, patient-to-patient transmission; **red arrows**, prior antibiotic use and antibiotic selection pressure driving resistance emergence; **purple arrows**, healthcare worker-to-patient transmission; **orange arrows**, environmental contamination and spread; **yellow arrows**, Medical devices (ventilators, central venous catheters, urinary catheters) serve as primary entry points for resistant organisms, particularly in neonatal and pediatric intensive care units.

Figure 3 provides an integrated view of how molecular resistance mechanisms operate alongside hospital-level risk factors to drive the burden of resistant infections in children. On the left, the figure recapitulates the key enzymatic, target-modification, efflux, and permeability mechanisms described above. On the right, it illustrates the hospital risk factors documented in the studies cited in this review: prior antibiotic use, which creates selection pressure favoring resistant organisms [23,26]; invasive medical devices such as central venous and urinary catheters, which serve as both infection entry points and independent predictors of mortality in pediatric Gram-negative bloodstream infections [9,10], patient-to-patient and healthcare-worker-to-patient transmission; and environmental contamination, all of which facilitate the horizontal spread of resistance determinants within and across units. The convergence of molecular mechanisms with healthcare-associated risk factors underscores the need for effective pediatric AMR control, which requires simultaneous intervention across multiple levels: stewardship, infection prevention, and diagnostic innovation.

### 2.3. Clinical Outcomes Associated with Resistant Infections

**Figure 4:**
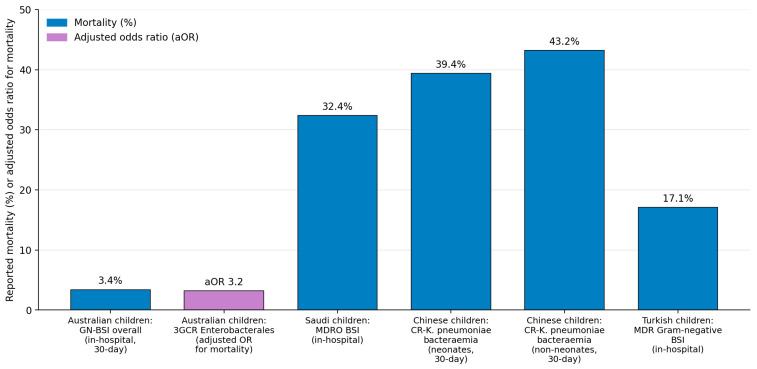
Reported mortality outcomes in pediatric infections by resistance phenotype. Values are presented exactly as reported in the source studies. aOR = adjusted odds ratio; BSI = bloodstream infection; CR = carbapenem-resistant; GN = Gram-negative; MDR = multidrug-resistant; MDRO = multidrug-resistant organism; 3GCR = third-generation cephalosporin-resistant [9,10,11,25].

Antibiotic resistance is consistently associated with worse clinical outcomes in children. Reported pediatric mortality outcomes from the validated studies in this review are shown in Figure 4.

Beyond mortality, resistant infections are associated with prolonged hospitalization, more frequent intensive care admission, and higher healthcare resource utilization. Pediatric patients with ESBL-producing UTIs experienced significantly longer total length of stay (β = 2.85 days; 95% CI 1.14–4.56), and longer ICU stays (β = 5.86 days; 95% CI 1.59–10.12) compared with non-ESBL UTIs [23]. At a population level, the GBD 2021 AMR analysis estimated that without intervention, AMR could be associated with up to 8.22 million deaths globally per year by 2050; a “better care” scenario incorporating improved infection control and access to appropriate antibiotics could avert 92.0 million cumulative deaths between 2025 and 2050 [2].

### 2.4. Vaccination as a Pillar of Antimicrobial Resistance Prevention

Vaccination is included in this review not merely as a strategy for preventing infectious disease, but because it operates as a direct and measurable upstream determinant of antimicrobial resistance. Vaccines prevent infections that would otherwise trigger antibiotic prescriptions, thereby reducing selection pressure on commensal and pathogenic flora. When vaccines specifically target serotypes enriched for resistance determinants, they also reduce the circulating reservoir of resistant strains. Both pathways directly shape the resistance landscape in pediatric populations and are therefore central to any comprehensive discussion of pediatric AMR.

**Figure 5:**
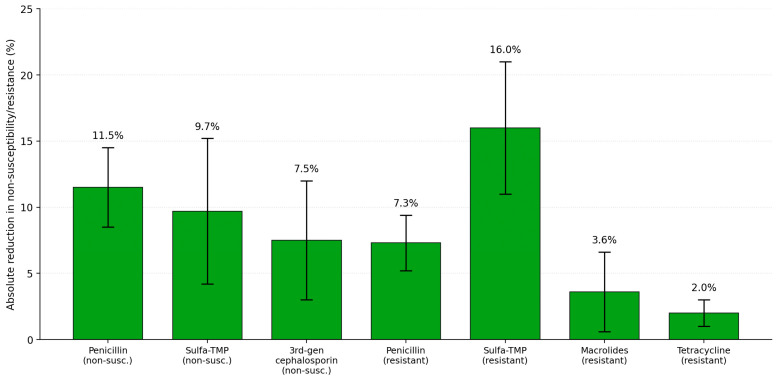
Impact of pneumococcal conjugate vaccines on antimicrobial non-susceptibility and resistance in pediatric pneumococcal isolates: pooled absolute reductions over 10 years post-implementation. Bars represent pooled point estimates, and whiskers represent 95% confidence intervals. Adapted from [46].

A meta-regression of 559 studies and 312,783 pediatric pneumococcal isolates demonstrated that, after pneumococcal conjugate vaccines (PCV) implementation, the proportion of pneumococci showing penicillin non-susceptibility fell by an absolute 11.5% (95% CI 8.6–14.4), penicillin resistance by 7.3% (95% CI 5.3–9.4), and sulfamethoxazole–trimethoprim non-susceptibility by 9.7% (95% CI 4.3–15.2) [46]. These pooled absolute reductions are shown in Figure 5. The clinical implication is that PCV programs serve as both infection-prevention and stewardship tools. Crucially, these are not merely reductions in infection burden. They represent measurable contractions in the pool of antibiotic-resistant pneumococcal clones circulating within pediatric communities.

Direct surveillance data reinforce this resistance-specific effect. In a 6-year active surveillance program in Mongolia following PCV13 introduction in 2016, von Mollendorf and colleagues documented that the proportion of nasopharyngeal samples from hospitalized children containing any AMR gene fell from 92.3% in the pre-PCV13 period to 85.3% after introduction (adjusted odds ratio [aOR] 0.49; 95% CI 0.34–0.70), and the proportion harboring three or more AMR genes fell from 46.8% to 27.6% (aOR 0.44; 95% CI 0.36–0.55) [47]. This finding demonstrates that vaccination directly reduces the AMR gene burden in hospitalized children, with immediate clinical and epidemiological relevance to the resistance outcomes discussed throughout this review.

The resistance-reducing impact of vaccines extends beyond *Streptococcus pneumoniae*. In England, a national live attenuated influenza vaccine (LAIV) program for young children was associated with a significant reduction in community antibiotic prescribing: every 10% increase in LAIV uptake in children aged 2–3 years was associated with a 2.7% reduction in respiratory tract infection antibiotic prescribing rates (95% CI 2.1–3.4%; *p* < 0.0001) [48]. Since antibiotic prescribing volume is the primary driver of selection pressure for resistance, this quantified reduction in prescribing translates directly into reduced resistance emergence.

### 2.5. Antimicrobial Stewardship in Pediatric Practice

Antimicrobial stewardship programs are included in this review because they represent the most direct institutional intervention available to reduce antibiotic selection pressure and thereby limit the emergence and spread of resistance. Multiple systematic reviews have demonstrated that pediatric antimicrobial stewardship programs (ASPs) produce measurable reductions in MDR organism rates and bacterial resistance prevalence, in addition to their well-documented effects on antibiotic consumption.

A systematic scoping review by Donà and colleagues, encompassing 113 studies of pediatric ASPs across all settings globally, found that seven studies quantified resistance outcomes directly: these studies demonstrated increased bacterial susceptibility, decreased prevalence of ESBL-producing organisms, and reduced rates of carbapenem resistance following ASP implementation, changes attributable to observed reductions in days of antimicrobial therapy [49]. This evidence positions ASP not merely as a prescribing-quality intervention but as a direct driver of the resistance trends documented throughout this review.

Evidence from LMICs is particularly compelling. A systematic review by Abo and colleagues of 34 antimicrobial stewardship intervention studies in children across 17 LMICs, representing more than 5 million patients, found that antimicrobial stewardship interventions resulted in significantly decreased rates of clinical infection (4/4 studies reporting this outcome), reduced clinical failure (2/2 studies), and most directly relevant to this review, a reduction in MDR organism colonization rates in all four studies that measured this endpoint [50]. Critically, no concomitant increase in mortality or length of stay was observed, refuting concerns that stewardship-driven reductions in antibiotic use might compromise patient safety. These data confirm that the resistance burden documented across the LMICs examined in Section 2.1, where MDR organism colonization rates are highest, is directly amenable to modification through ASP implementation.

Antimicrobial stewardship is the most consistently effective institutional response to pediatric AMR. A 30-month prospective audit with feedback ASP at a U.S. children’s hospital reviewed 10,460 broad-spectrum or selected antibiotic courses, with clinicians complying with stewardship recommendations in 92% of cases [51].

Compared with a control group of 25 similar children’s hospitals, the intervention was associated with a 7% monthly decline in days-of-therapy and an 8% decline in length-of-therapy per 1000 patient-days, with even larger declines (17% and 18%, respectively) for the broad-spectrum agents specifically targeted by the program [51]. Reductions in this magnitude in targeted antibiotic use directly diminish the selection pressure that drives the emergence of the ESBL-producing Enterobacterales and carbapenem-resistant phenotypes documented in Section 2.1. More recent multicenter evidence from Peru strengthens the evidence base for pediatric antimicrobial stewardship implementation in resource-limited settings. In a cohort of 1250 hospitalized children with bloodstream or urinary tract infections, greater maturity of the antimicrobial stewardship program was independently associated with lower rates of ESBL-producing Enterobacteriaceae infections. Direct antimicrobial stewardship intervention for pediatric bloodstream and urinary tract infections was associated with a median reduction of three hospital days, shorter time to appropriate therapy, and a 4.2% reduction in 30-day mortality after propensity-score matching analysis [30]. Programmatic recommendations and quality metrics for pediatric ASPs are summarized in Table 2.

### 2.6. Novel Therapeutics for Pediatric Multidrug-Resistant Infections

Several β-lactam/β-lactamase-inhibitor combinations and novel agents have been developed for MDR Gram-negative infections; however, pediatric data remain limited. A recent narrative review [33] summarized the available pediatric pharmacological and clinical evidence for ceftolozane–tazobactam, ceftazidime–avibactam, meropenem–vaborbactam, imipenem–cilastatin–relebactam, and the siderophore cephalosporin cefiderocol, noting that several of these agents lack pediatric indications approved by the U.S. Food and Drug Administration and the European Medicines Agency, leading to uncertain pediatric-specific dosing strategies. None of these agents reliably covers metallo-β-lactamase (MBL)-producing organisms, an important therapeutic gap in regions with high NDM prevalence [32,33]. For metallo-β-lactamase-producing organisms, aztreonam combined with avibactam is in clinical development. Selected agents and their pediatric status are summarized in Table 3.

## 3. Discussion

The synthesis of validated evidence reveals a complex and rapidly evolving pediatric AMR landscape. Several observations emerge consistently across the studies surveyed in this review. The findings reviewed also suggest that AMR should be viewed as an ecological problem rather than solely an infectious disease issue.

First, resistant Gram-negative infections, especially those caused by ESBL-producing and carbapenem-resistant Enterobacterales, are independently associated with substantially worse outcomes in children, including higher mortality, prolonged hospitalization, and more frequent intensive care admissions [9,11,23,25]. The dominance of CTX-M-type ESBLs, particularly CTX-M-15, across continents reflects the global dissemination of high-risk clones [9].

Second, prior antibiotic exposure is a dominant patient-level driver of subsequent resistance, both in invasive infections and in the commensal microbiome [8,23,26]. National outpatient prescribing data show that as much as one-third of pediatric outpatient antibiotic prescribing is unnecessary [7], and meta-analytic data link recent antibiotic prescriptions to a 13-fold increase in pediatric urinary *E. coli* resistance for up to 6 months [24]. Pediatric resistance patterns are incredibly influenced by exposure to antibiotics that are outside the realm of their infection; further, this repeated antibiotic administration during infancy to childhood leads to alterations in the intestinal microbiome during a critical developmental period [8,24] of their lives, allowing for reduced microbial diversity and natural selection amongst bacteria with antibiotic resistance mechanisms.

Third, the global distribution of resistance is highly inequitable. The heaviest burden falls on LMICs whose surveillance and stewardship infrastructure is least developed [12,13,14]. The OECD–non-OECD disparity in pediatric *E. coli* UTI resistance shown in Figure 1 is striking, but it likely understates the total clinical impact because diagnostic capacity in LMICs is also limited.

Fourth, mechanistic and clinical heterogeneity argue against one-size-fits-all empiric strategies. The geographic dominance of different carbapenemase types (KPC, NDM, OXA-48-like) means that newer β-lactam/β-lactamase-inhibitor combinations vary in their utility across regions [32,33]. Empiric protocols, therefore, need to be calibrated to local resistance epidemiology and updated as surveillance data mature. The Australian experience demonstrates that comprehensive, prospectively coordinated pediatric surveillance can yield actionable, age-stratified estimates that inform such protocols [4,9].

Fifth, the GBD 2021 AMR analysis showed that AMR mortality in children younger than five years has fallen by more than 50% globally over three decades, evidence that infection prevention, vaccination, and improved access to care can move the needle [2]. The pediatric pneumococcal experience reinforces this point: PCV introduction was associated with absolute reductions in penicillin and sulfamethoxazole–trimethoprim resistance of 7% or more and up to 16%, respectively, over a decade [46]. Vaccines should therefore be regarded as front-line AMR prevention, not as adjuncts to it.

Sixth, there is an increasing convergence between community-associated and healthcare-associated resistance. Previously, MDR pathogens were confined to hospital settings such as the ICU or PICU, but these studies have demonstrated that resistant organisms have become established in community settings over time [17,22,24]. In turn, clinicians are no longer able to rule out that community-acquired infections are solely susceptible to narrow-spectrum first-line agents [24]. Instead, many feel more comfortable prescribing empiric regimens rather than “missing” coverage of the bug, especially in younger patients who have not had a chance to build up their immune systems. In turn, this continues to push the one-size-fits-all empirical regimen narrative and, over time, increases antibiotic resistance.

Finally, antimicrobial stewardship is the most consistently effective institutional response, but its impact depends on integration with diagnostic stewardship, infection prevention and control, and vaccination. Rapid molecular diagnostics shorten the empiric-therapy window during which broad-spectrum antibiotics drive resistance [52], while infection prevention, especially careful management of central venous and urinary catheters, directly addresses the device-related risk demonstrated in single-center pediatric Gram-negative cohorts [9,10].

## 4. Critical Analysis and Research Gaps

### 4.1. Lack of Pediatric-Specific Clinical Trials

A persistent limitation is the scarcity of randomized controlled trials specifically designed for children with multidrug-resistant infections. Most data on novel antibiotics derive from adult populations, leading to off-label use, age-extrapolated dosing, and uncertain pediatric safety profiles [3,33]. Pediatric pharmacokinetic and pharmacodynamic data are particularly limited for critically ill children, neonates, and obese children, the very populations most likely to receive novel agents.

Additionally, another limitation is that the pediatric population is often grouped into broad age categories despite profound physiological differences [3,67]. Categorizing neonates, infants, children, and adolescents aged 0 to 18 years together may reduce the precision of dose recommendations, obscuring age-specific toxicity profiles for other antimicrobial agents and increasing the risk of reinfection or resistance. Furthermore, this lack of pediatric-specific clinical trials limits the understanding of optimal treatment durations for resistant infections in children, as current recommendations are adapted from adult guidelines [33] and practice, even though prolonged antimicrobial exposure can have various developmental and microbiome-related consequences during childhood.

Another major issue that warrants greater discussion is the developmental specificity of antimicrobial resistance in the pediatric population. Children’s physiologic makeup alters both their infection susceptibility and their antibiotic pharmacokinetics, and their physiologic makeup cannot be compared to that of an “adult body”. To delve deeper, neonates, particularly preterm infants, have immature innate and adaptive immune responses [67] due to limited or absent exposure to the environment and pathogens. Along with their reduced neutrophil function, altered complement activity, and highly permeable mucosal barriers, all factors that increase their vulnerability to invasive organisms, renal clearance and hepatic metabolism change rapidly across infancy and childhood [18]. This complicates antibiotic dosing, as there is a larger margin for under- or overexposure, and providers are forced to extrapolate from adult studies [3,33] to create a proper regimen for pediatric patients, even though physiologic differences between pediatric and adult patients differ. These developmental variables create a narrow therapeutic window in which empiric therapy can fall short, leading to inadequate antibiotic coverage whilst also leaving room for antibiotic resistance.

Resistant infections open the door for neurodevelopmental disruption through prolonged hospitalization during critical developmental periods, repeated healthcare exposure throughout childhood, and increased emotional disturbances and stress that lead to diminished quality of life.

### 4.2. Geographic and Methodological Imbalances

Despite extensive surveillance in some high-income settings, longitudinal pediatric resistance data from Sub-Saharan Africa, the Middle East, and parts of Asia remain limited [12,13,15,16]. Many published estimates are derived from single-center retrospective studies, which limit the generalizability of regional resistance estimates [10,11]. The BARNARDS network demonstrated what is possible with prospective multi-country surveillance in LMICs [14], but such infrastructure remains the exception rather than the norm. Methodological differences across studies also complicate comparisons between regions, as variability in resistance definitions, laboratory susceptibility standards, and inclusion criteria and outcome measures limit the ability to synthesize pediatric antimicrobial resistance data globally. Future surveillance efforts would also benefit from standardized pediatric AMR reporting frameworks that are inclusive across various settings, such as community, outpatient, and rural hospitals, rather than being limited to inpatient settings.

### 4.3. Microbiome and Long-Term Ecological Consequences

Although it is well established that early antibiotic exposure [8,24] shapes the developing microbiome and the resistome, the long-term clinical consequences in children, including the persistence of resistance genes, effects on later infection susceptibility, and broader immunological outcomes, remain incompletely characterized. Longitudinal pediatric cohort studies linking microbiome composition with infection outcomes are needed. An important unresolved question is how long antibiotic-induced microbiome disruption persists following an exposure during early childhood. Evidence suggests that resistant organisms and genes may remain detectable months after treatment [24], but this duration remains uncertain in pediatric data. Furthermore, this relationship between microbiome disruption and later noninfectious disease warrants further investigation. Early-life antibiotic exposure has been linked to increased risk of hypersensitivity, obesity, and metabolic dysregulation [68,69], suggesting that antimicrobial stewardship could have benefits extending beyond resistance prevention. All in all, these topics warrant thorough research and analysis to provide a comprehensive overview of the long-term consequences of acquiring antibiotic resistance at a young age.

### 4.4. Diagnostic Implementation Gaps

While the analytical performance of rapid molecular diagnostics is increasingly well documented, evidence on how to scale and integrate these tools into routine pediatric care, particularly in resource-constrained settings, is limited [52]. Moreover, although rapid molecular diagnostics also shorten time to pathogen identification, implementation barriers remain substantial in many pediatric healthcare systems. The cost of equipment, limited staffing, and inadequate staff training continue to restrict the use of these diagnostics, particularly in LMICs. Another challenge is that most current studies tend to focus on diagnostics from a metrics standpoint rather than a patient-centered perspective that considers mortality rates, hospital length of stay, antibiotic exposure duration, etc. Thus, studies linking diagnostic intervention to changes in pediatric prescribing and outcomes should be a research priority.

### 4.5. Stewardship Outside Tertiary Centers

A major evidence gap is that most published pediatric ASP evidence comes from tertiary children’s hospitals [51]. However, pediatric outpatient settings account for a substantial proportion of antibiotic exposure, including respiratory tract infections, otitis media, and other viral infections, all of which can drive unnecessary antibiotic overprescribing. Another underexplored area is the role of behavioral and communication interventions in reducing pediatric overprescribing. Patient education, approaches to delayed administration, and adherence may substantially influence antibiotic use outside hospital settings. Research is also limited regarding antibiotic stewardship implementation in rural or underdeveloped healthcare systems, and overall, comparative-effectiveness research on stewardship in community pediatric settings, primary care, and resource-limited environments is relatively sparse, leaving important gaps in generalizable implementation guidance [6].

## 5. Future Directions

Addressing pediatric AMR will require coordinated, multilevel action. Specific priorities include the following:

### 5.1. Scale Pediatric-Focused Antimicrobial Stewardship

Mature ASPs combining prospective audit with feedback, AWaRe-based prescribing metrics, and outpatient stewardship should be a priority for pediatric services globally, with explicit attention to neonatal intensive-care units, oncology services, and other high-risk settings [6,26,51]. Stewardship programs should also consider incorporating age-specific prescribing guidelines, as antimicrobial selection, dosing, and toxicity risks differ across pediatric populations. Embedding stewardship principles into routine pediatric training curricula may also help improve long-term tendencies toward overprescribing among future healthcare providers and reduce the habit of prescribing unnecessarily broad empiric therapies.

### 5.2. Expand Rapid Molecular Diagnostics, Especially in LMICs

Allowing greater access to rapid diagnostics may play a significant role in neonatal and pediatric intensive care settings, where delays in effective therapy are strongly associated with worse outcomes. Investment in laboratory capacity and the deployment of point-of-care molecular tests can accelerate the transition from broad-spectrum empiric therapy to targeted treatment [12,52]. With this diagnostic expansion, physician/clinician training and stewardship integration must also be considered to help ensure that these rapid molecular diagnostic results truly translate into practice.

### 5.3. Integrate Whole-Genome Sequencing into Surveillance

Genomic surveillance, as exemplified by the Australian pediatric Gram-negative bloodstream program and the BARNARDS LMIC neonatal sepsis network, can identify clonal spread, emerging resistance determinants, and high-risk lineages, supporting targeted public health responses [9,14]. This integration of genomic data with clinical and epidemiological surveillance may help improve understanding of how resistance determinants spread between community and healthcare settings. International genomic data-sharing networks could further help detect emerging high-risk pediatric patients early and support global public health responses. Despite the various benefits of integrating whole-genome sequencing into surveillance, its routine use remains limited by cost, limited expertise, and accessibility.

### 5.4. Conduct Pediatric-Inclusive Clinical Trials

Future pediatric antimicrobial trials should prioritize the inclusion of pediatric populations, particularly neonates, immunocompromised children, and critically ill patients, who are often excluded from studies, to obtain comprehensive data on antibiotic resistance across all pediatric patients. Children should be included in early-phase trials of novel antimicrobials wherever ethically and pharmacologically feasible, and comparative-effectiveness studies for MDR infections in children are urgently needed [3,33]. Moreover, long-term follow-up studies should be performed to evaluate the developmental and toxicological consequences of exposure to antibiotics in childhood, such as five to even ten years after treatment.

### 5.5. Strengthen Infection Prevention and Control

Careful management of invasive devices, particularly central venous and urinary catheters, remains a cornerstone of preventing healthcare-associated transmission of resistant pathogens [9,10]. Strict hand hygiene, environmental cleaning, and contact precautions remain essential for reducing the transmission of resistant organisms within and across hospital units. Further, routine surveillance for asymptomatic colonization with multidrug-resistant organisms may help identify high-risk patients and prevent hospital outbreaks. Efforts such as these can strengthen preventive measures against infection, thereby reducing the need for antibiotics and, consequently, antibiotic resistance.

### 5.6. Use Vaccines as Primary AMR Prevention

Vaccinations not only help prevent infections but also reduce overall antibiotic exposure. Maintaining childhood vaccination and continuing vaccination throughout adulthood is especially important in areas with limited antimicrobial stewardship, where prevention may be more feasible than altering prescribing practices. PCV introduction has produced measurable absolute reductions in pediatric pneumococcal antibiotic resistance globally [46]; expanding access to existing vaccines and developing vaccines against other priority MDR pathogens (e.g., ESBL *E. coli*, *K. pneumoniae*) is a key long-term strategy [2]. Thus, indirect herd protection through vaccination strategies reduces the transmission of these resistant organisms within unvaccinated or vulnerable populations.

### 5.7. Address Health-System Inequities

To reduce pediatric antimicrobial resistance, disparities in the structural determinants of health must also be addressed. Sustained progress in LMICs will require investment in microbiology infrastructure, vaccine access, and workforce training, complemented by international cooperation. The ‘better care’ modeling scenario in the GBD 2021 AMR analysis suggests that approximately 92 million cumulative deaths could be averted globally between 2025 and 2050 with improved infection control and access to appropriate antibiotics [2]. International partnerships and funding can help to support a sustainable workplace, training, and ensure equitable access to the highest quality of care. Without these measures, advances in healthcare and other diagnostics remain disproportionate, creating an even larger and disproportionate gap between the quality of patient care and patient education.

## 6. Materials and Methods

### 6.1. Literature Search Strategy

A comprehensive literature review was conducted using PubMed/MEDLINE and Google Scholar, supplemented by targeted searches of indexed peer-reviewed journals. Search terms combined Medical Subject Headings (MeSH) and free-text keywords, including “antibiotic resistance,” “antimicrobial resistance,” “pediatric,” “children,” “neonatal,” “multidrug-resistant,” “ESBL,” “carbapenem resistance,” “MRSA,” “stewardship,” “AWaRe,” “pneumococcal conjugate vaccine,” and pathogen-specific terms.

This article is a narrative review and was not conducted as a systematic review; accordingly, the search was not exhaustive and did not follow PRISMA methodology. PubMed/MEDLINE served as the primary database, and Google Scholar was used as a supplementary source to capture additional records, including surveillance and gray-literature reports not indexed in MEDLINE. The reference lists of key articles were screened manually. The record counts reported below are approximate and are provided for transparency rather than as a reproducible systematic-search yield.

### 6.2. Inclusion and Exclusion Criteria

Studies were eligible for inclusion if they met all of the following criteria: (1) reported original data or systematic synthesis on antibiotic resistance, mechanisms, clinical outcomes, or interventions in pediatric populations (defined as 0–18 years, including neonates); (2) appeared in a peer-reviewed journal indexed in PubMed/MEDLINE’ Google Scholar, or in a formally indexed surveillance report; (3) were published between January 2014 and February 2026, with foundational older primary studies (≤2014) retained when they provided primary data not superseded by newer work; and (4) were available in English, or had a translated abstract sufficient to verify primary findings.

Studies were excluded if they (1) reported only adult data with no pediatric stratification; (2) were preprints not subsequently published in peer-reviewed form; (3) were narrative commentaries, editorials, or opinion pieces without primary or systematically reviewed data; (4) reported on viral, fungal, parasitic, or mycobacterial resistance unless directly relevant to bacterial AMR co-management; or (5) had a citation that could not be independently verified against PubMed metadata or the publisher record.

### 6.3. Study Identification and Selection

The initial PubMed/MEDLINE and Google Scholar search returned approximately 1840 candidate records. After removal of duplicates and screening of titles and abstracts against the inclusion and exclusion criteria, 187 full-text articles were assessed for eligibility. Following full-text review, 69 unique sources met all inclusion criteria and are cited in this manuscript. The remaining records were excluded for the following reasons: adult-only or non-pediatric stratification, insufficient methodological detail, unverifiable citation or non-indexed source, preprints without subsequent peer-reviewed publication, or content superseded by a more recent or higher-quality source.

### 6.4. Data Synthesis

Given the heterogeneity in study designs, populations, geographic settings, and outcome measures, a narrative synthesis approach was used. Quantitative findings (resistance prevalence, mortality, length of stay) are reported as published, with explicit attribution to the specific source study. No new pooled estimates or meta-analytic summaries were generated. Figures and tables in this manuscript display only data values from the cited primary or systematic review sources.

We acknowledge the principal limitations of this approach: reliance on two literature sources may have missed studies that were indexed elsewhere, and narrative synthesis does not permit formal quantitative pooling or a structured risk-of-bias assessment. These limitations are weighed against the explicit aim of the review, which is to provide an integrated, cross-cutting synthesis of pediatric antimicrobial resistance, spanning epidemiology, mechanisms, outcomes, and management, that a narrowly scoped systematic review of a single sub-topic could not deliver.

## 7. Conclusions

Pediatric antimicrobial resistance stands at the intersection of microbiology, clinical medicine, and global health equity. In line with its stated objective, this review has synthesized the epidemiology, mechanisms of resistance, clinical outcomes, and management of antimicrobial resistance across the full pediatric age range, from neonates to adolescents. This review has documented a landscape in which multidrug-resistant Gram-negative and Gram-positive pathogens impose a measurable and disproportionate mortality burden on children, particularly neonates and those in LMICs, while the diagnostic, therapeutic, and stewardship tools available to clinicians lag substantially behind those developed for adults. At the same time, the evidence affirms that this trajectory is not fixed: pneumococcal conjugate vaccines have produced demonstrable reductions in pediatric pneumococcal antimicrobial resistance, and global AMR mortality in children younger than 5 years of age has fallen by more than 50% since 1990, illustrating that coordinated public health investment can alter the course of resistance. Sustaining and accelerating that progress will require expanding pediatric stewardship programs, integrating genomic surveillance into clinical and public health practice, urgently addressing geographic and demographic gaps in clinical trial enrollment, and strengthening infection prevention infrastructure across all settings. Ultimately, protecting children from the harms of antimicrobial resistance is not solely a scientific challenge, but it is a matter of equity, demanding that the same rigor and resources applied to adult infectious disease be extended, without delay, to the most vulnerable patients.

## Figures and Tables

**Table 1:** Pathogen-specific resistance findings in pediatric populations from the studies cited in this review. Values are reproduced as published in the original sources.

Pathogen/Phenotype	Setting/Population	Reported Finding	Reference
3GCR Enterobacterales (Gram-negative BSI)	5 Australian children’s hospitals (2019–2021); 931 episodes, 818 children	22% (138/630) of Enterobacterales isolates resistant to 3GC; *bla_CTX-M-15_* is the most common ESBL gene (36%); aOR 3.2 (95% CI 1.6–6.4) for mortality with 3GCR	[9]
3GCR and ESBL Enterobacterales (national surveillance)	U.S. national surveillance, 1999–2011; 368,398 pediatric isolates	3GCR prevalence rose from 1.39% (1999–2001) to 3.0% (2010–2011); ESBL phenotype from 0.28% to 0.92%; 74% of ESBL isolates were resistant to ≥3 antibiotic classes	[22]
ESBL-producing *E. coli*/*K. pneumoniae* (UTI)	Tertiary center, Taiwan (2017–2021); 327 hospitalized children	ESBL prevalence 14.1%; recent antibiotic exposure within 6 months and preterm gestational history were independent risk factors; longer length of stay (β 2.85 days) and ICU stay (β 5.86 days)	[23]
*E. coli* pediatric UTI (community-acquired)	Systematic review/meta-analysis, 58 studies, 77,783 isolates	OECD vs. non-OECD pooled resistance: ampicillin 53.4% vs. 79.8%; co-amoxiclav 8.2% vs. 60.3%; ciprofloxacin 2.1% vs. 26.8%; nitrofurantoin 1.3% vs. 17.0%	[24]
Carbapenem-resistant *K. pneumoniae*	Single-center, China (2018–2021); 70 children	30-day mortality 39.4% in neonates, 43.2% in older children; appropriate targeted therapy associated with reduced mortality	[25]
MRSA/Carbapenem-resistant GNB/VRE	Multinational pediatric oncology/HSCT cohort (2015–2017); 1031 patients, 1291 BSI episodes	17% methicillin resistance in *S. aureus*; 9% meropenem resistance in Gram-negatives; 40% vancomycin resistance in *E. faecium*; prior carbapenem exposure associated with resistant Gram-negative BSI	[26]
Multi-drug-resistant Gram-negatives in neonatal sepsis	BARNARDS network, 7 LMICs, 36,285 neonates enrolled, 916 isolates sequenced	*K. pneumoniae* leading sepsis pathogen; isolates harbored multiple cephalosporin and carbapenem resistance genes; all isolated pathogens were resistant to multiple antibiotic classes, including those used in neonatal sepsis	[14]
Pediatric Gram-negative sepsis in LMICs	Systematic review, 30 studies, 71,326 children (Asia and Africa)	Neonatal *K. pneumoniae* median resistance: ampicillin 94% (Asia)/100% (Africa); cephalosporins 84% (Asia)/50% (Africa); MDR Salmonella spp. median 30% (IQR 0–59.6) Asia, 75% (IQR 30–85.4) Africa	[13]
MDR organisms (overall)	Tertiary hospital, Saudi Arabia (2021–2022); pediatric cultures	MDROs in 42% of patients with positive cultures; *K. pneumoniae* most common (39.5% of MDR cultures); 32.4% mortality with MDROs vs. 3.9% without	[11]
MDR Gram-negative BSI	Single-center, Turkey (2022); 102 children, 123 cultures	28.5% MDR among isolates; 17.1% mortality in resistant cases vs. 10.5% overall; urinary catheter independent predictor of mortality (OR 5.68)	[10]
MDR Salmonella Typhi (H58)	Febrile children, Nairobi County, Kenya; 120 isolates	50% MDR; 65.6% ampicillin resistance; 67.6% with QRDR mutations conferring reduced ciprofloxacin susceptibility	[15]
Invasive MRSA in children	U.S. population-based surveillance, 2005–2010; 876 cases	Estimated invasive MRSA incidence 43.9/100,000 in infants <90 days vs. 2.0/100,000 in older children; CA-MRSA incidence rose 10.2% per year (95% CI 2.7–18.2)	[27]
Macrolide-resistant Mycoplasma pneumoniae pneumonia	Meta-analysis, 11 studies, 1143 children (East Asia)	Tetracyclines superior to macrolides for fever duration (WMD 1.64 days), hospital stay (WMD 1.22 days), and therapeutic efficacy (OR 0.33 for macrolide vs. tetracycline)	[28]
Amoxicillin-resistant oral bacteria	Children aged 4–5 years (community); 40 children, 224 isolates	100% carriage; median resistant proportion 2.4% without recent amoxicillin vs. 10.9% after recent use (*p* < 0.01); 65% of resistant isolates were also resistant to at least one of three antibiotics: penicillin/erythromycin/tetracycline	[8]
Pediatric AMR (general)	Tertiary pediatric hospital, Romania, 1-year retrospective; 1445 isolates	Range of resistance phenotypes, including ESBL-producing Gram-negatives and MRSA in pediatric inpatient isolates	[29]
Pediatric AMR in a conflict setting	Damascus Hospital, Syria; 116 children, 177 cultures	Most prevalent organisms: *S. aureus* (33%), *Enterobacter* (21%); highest resistance to 3GC and ceftriaxone (70% use); 51% nosocomial infections; 16% mortality	[16]

3GCR = third-generation cephalosporin-resistant; aOR = adjusted odds ratio; BSI = bloodstream infection; CA-MRSA = community-associated methicillin-resistant *Staphylococcus aureus*; HSCT = hematopoietic stem-cell transplant; LMIC = low- and middle-income country; MDR = multidrug-resistant; MDRO = multidrug-resistant organism; MRSA = methicillin-resistant *Staphylococcus aureus*; OR = odds ration; QRDR = quinolone resistance-determining region; UTI = urinary tract infection; VRE = vancomycin-resistant *enterococcus*; WMD = weighted mean difference.

**Table 2:** Pediatric antimicrobial-stewardship strategies and their supporting evidence from validated peer-reviewed sources.

Stewardship Strategy	Evidence (Validated Source)	Implementation Note
Prospective audit with feedback	30-month quasi-experimental study at a U.S. children’s hospital reviewing 10,460 antibiotic courses; 92% compliance with recommendations; 17–18% decline in days-of-therapy and length-of-therapy per 1000 patient-days for selected antibiotics [51].	Requires dedicated ID pharmacist and physician time; sustainable in tertiary children’s hospitals.
AWaRe-classification monitoring	1-day point-prevalence surveys in 56 countries (23,572 patients) demonstrated wide global variation in Access vs. Watch vs. Reserve antibiotic use in hospitalized children [6]; ARPEC global PPS in 73 hospitals showed pediatric and neonatal antibiotic-use rates significantly higher in non-European hospitals than European hospitals [5].	Useful as a simple traffic-light metric for tracking appropriateness over time and benchmarking institutions.
Outpatient diagnostic and prescribing stewardship	U.S. national analysis (NAMCS/NHAMCS 2010–2011) estimated that of 506 outpatient antibiotic prescriptions per 1000 population, only 353 were appropriate; respiratory tract infections accounted for the largest share of inappropriate prescribing [7]. Routine antibiotic prescribing in primary care increases the odds of subsequent resistance by up to 13.2-fold for up to six months [24].	Targets respiratory infections, otitis media, pharyngitis; can leverage parent-facing communication training and clinical decision support.
ASP impact in resource-limited pediatric settings	Asia-Pacific narrative review [12] documents successful local ASP and infection-prevention initiatives that reduced antibiotic overuse in specific settings, while emphasizing infrastructure barriers in many LMICs.	Implementation must be tailored to local microbiology capacity and antibiotic supply chains.
ASP impact in resource-limited pediatric settings	The Peruvian multicenter study [30] suggests that institutional stewardship maturity can significantly influence both resistance prevalence and patient outcomes.	Investments in stewardship infrastructure may yield measurable reductions in ESBL burden, mortality, and hospital resource utilization.
Diagnostic stewardship and rapid molecular testing	A recent narrative review of metagenomic next-generation sequencing and machine-learning–assisted susceptibility prediction in pediatric infections highlights the potential to shorten time to targeted therapy, while noting genotype–phenotype discordance and implementation barriers [52].	Most useful when paired with a prospective audit so genotype-driven recommendations reach clinicians in real time.

ASP = antimicrobial stewardship program; AWaRe = WHO Access, Watch, Reserve classification; ID = infectious diseases; LMIC = low- and middle-income country; NAMCS = National Ambulatory Medical Care Survey; NHAMCS = National Hospital Ambulatory Medical Care Survey; PPS = point-prevalence survey.

**Table 3:** Selected novel β-lactam and β-lactam/β-lactamase-inhibitor combinations reviewed for pediatric multidrug-resistant Gram-negative infections.

Agent	Class/Mechanism	Spectrum (Per Cited Reviews)	Pediatric Status (Per Cited Reviews)
Ceftazidime–avibactam	3rd-gen cephalosporin + non-β-lactam β-lactamase inhibitor	ESBL-, AmpC-, KPC- and OXA-48-like-producing Enterobacterales; not active vs. MBLs	Pediatric pharmacokinetic data and use [33,53,54,55,56]
Ceftolozane–tazobactam	Antipseudomonal cephalosporin + β-lactamase inhibitor	MDR *Pseudomonas aeruginosa*, ESBL Enterobacterales	Pediatric PK data [33,57,58,59]
Meropenem–vaborbactam	Carbapenem + boronate β-lactamase inhibitor	KPC-producing Enterobacterales; not active vs. MBLs	Pediatric data [33,60,61,62]
Imipenem–cilastatin-relebactam	Carbapenem + diazabicyclooctane β-lactamase inhibitor	KPC-producing Enterobacterales, MDR *P. aeruginosa*	Limited pediatric data [33,63]
Cefiderocol	Siderophore cephalosporin	CRE including MBLs, MDR *P. aeruginosa*, *Acinetobacter baumannii*	Limited pediatric data [33,64,65,66]

Spectrum and pediatric-status descriptors are summarized from the cited literature. Specific FDA/EMA approvals and approved age ranges may have been updated since publication of those reviews; clinicians should consult current product labeling. CRE = carbapenem-resistant Enterobacterales; MBL = metallo-β-lactamase; MDR = multidrug-resistant; PK = pharmacokinetic.

## Data Availability

No new data were created or analyzed in this study. Data sharing is not applicable to this article.

## Abbreviations

The following abbreviations are used in this manuscript:

3GCR	Third-Generation Cephalosporin-Resistant
AMR	Antimicrobial Resistance
aOR	Adjusted Odds Ratio
ARPEC	Antibiotic Resistance and Prescribing in European Children
ASP	Antimicrobial Stewardship Program
AWaRe	Access, Watch, Reserve (WHO antibiotic classification)
BARNARDS	Burden of Antibiotic Resistance in Neonates from Developing Societies
BSI	Bloodstream Infection
CA-MRSA	Community-Associated Methicillin-Resistant *Staphylococcus aureus*
CI	Confidence Interval
cIAI	Complicated Intra-Abdominal Infection
CRE	Carbapenem-Resistant Enterobacterales
cUTI	Complicated Urinary Tract Infection
EMA	European Medicines Agency
ESBL	Extended-Spectrum β-Lactamase
FDA	U.S. Food and Drug Administration
GBD	Global Burden of Disease
GNBSI	Gram-Negative Bloodstream Infection
HAP	Hospital-Acquired Pneumonia
HSCT	Hematopoietic Stem-Cell Transplant
ICU	Intensive Care Unit
IQR	Interquartile Range
KPC	*Klebsiella pneumoniae* Carbapenemase
LMIC	Low- and Middle-Income Countries
MBL	Metallo-β-Lactamase
MDR	Multidrug-Resistant
MDRO	Multidrug-Resistant Organism
MeSH	Medical Subject Headings
MRSA	Methicillin-Resistant *Staphylococcus aureus*
NAMCS	National Ambulatory Medical Care Survey
NDM	New Delhi Metallo-β-lactamase
NHAMCS	National Hospital Ambulatory Medical Care Survey
OECD	Organisation for Economic Co-operation and Development
OR	Odds Ratio
OXA-48	Oxacillinase-48-type Carbapenemase
PCV	Pneumococcal Conjugate Vaccine
PICU	Pediatric Intensive Care Unit
PK	Pharmacokinetics
PPS	Point-Prevalence Survey
QRDR	Quinolone Resistance-Determining Region
UTI	Urinary Tract Infection
VAP	Ventilator-Associated Pneumonia
VRE	Vancomycin-Resistant Enterococcus
WGS	Whole-Genome Sequencing
WHO	World Health Organization
WMD	Weighted Mean Difference
